# Acenocoumarol Exerts Anti-Inflammatory Activity via the Suppression of NF-κB and MAPK Pathways in RAW 264.7 Cells

**DOI:** 10.3390/molecules28052075

**Published:** 2023-02-22

**Authors:** Hyun-Ju Han, Chang-Gu Hyun

**Affiliations:** Jeju Inside Agency and Cosmetic Science Center, Department of Chemistry and Cosmetics, Jeju National University, Jeju 63243, Republic of Korea

**Keywords:** acenocoumarol, drug repurposing, inflammation, macrophages, MAPK, NF-κB

## Abstract

The repurposing of already-approved drugs has emerged as an alternative strategy to rapidly identify effective, safe, and conveniently available new therapeutic indications against human diseases. The current study aimed to assess the repurposing of the anticoagulant drug acenocoumarol for the treatment of chronic inflammatory diseases (e.g., atopic dermatitis and psoriasis) and investigate the potential underlying mechanisms. For this purpose, we used murine macrophage RAW 264.7 as a model in experiments aimed at investigating the anti-inflammatory effects of acenocoumarol in inhibiting the production of pro-inflammatory mediators and cytokines. We demonstrate that acenocoumarol significantly decreases nitric oxide (NO), prostaglandin (PG)E_2_, tumor necrosis factor (TNF)-α, interleukin (IL)-6, and IL-1β levels in lipopolysaccharide (LPS)-stimulated RAW 264.7 cells. Acenocoumarol also inhibits the expression of NO synthase (iNOS) and cyclooxygenase (COX)-2, potentially explaining the acenocoumarol-induced decrease in NO and PGE_2_ production. In addition, acenocoumarol inhibits the phosphorylation of mitogen-activated protein kinases (MAPKs), c-Jun N terminal kinase (JNK), p38 MAPK, and extracellular signal-regulated kinase (ERK), in addition to decreasing the subsequent nuclear translocation of nuclear factor κB (NF-κB). This indicates that acenocoumarol attenuates the macrophage secretion of TNF-α, IL-6, IL-1β, and NO, inducing iNOS and COX-2 expression via the inhibition of the NF-κB and MAPK signaling pathways. In conclusion, our results demonstrate that acenocoumarol can effectively attenuate the activation of macrophages, suggesting that acenocoumarol is a potential candidate for drug repurposing as an anti-inflammatory agent.

## 1. Introduction

Inflammation is the complex biological response of the immune system and can be triggered as a part of a defensive mechanism by a variety of factors such as cell damage, irritants, pathogens, toxins, and other compounds; this is characterized by redness, swelling, fever, pain, and impaired function at the tissue level [1,2]. Although the inflammatory response is an important mechanism for host defense, chronic inflammation is the underlying cause of several diseases including asthma, endometriosis, obesity, atherosclerosis, rheumatoid arthritis (RA), and psoriasis [3,4,5]. Therefore, controlling abnormal inflammatory responses is a vital tool for the prevention and treatment of inflammatory diseases [6].

Macrophages are central players in systemic inflammation, associated with meta-inflammation and inflammaging. Over the years, several experimental models have been designed to facilitate the development of novel anti-inflammatory drugs, although in vitro models of RAW 264.7 cells are now the most widely used [7]. Mouse macrophage RAW264.7 is a functional macrophage line, transformed by the *Abelson leukemia* virus, that requires LPS for full activation. There is a consensus that inflammatory mediators and pro-inflammatory cytokines should initially be measured to screen for possible anti-inflammatory effects. More specifically, pro-inflammatory cytokines such as tumor necrosis factor (TNF)-α and interleukins (IL)-1β and IL6 as well as inflammatory mediators, together with NO and PGE_2_, should be measured. Therefore, the LPS stimulation of RAW 264.7 macrophages is commonly used as a classical inflammatory cell model to evaluate the anti-inflammatory activity and the mechanisms underlying the action of drugs [7,8,9]. There are four main categories of signaling pathways, whose activation during inflammation leads to the secretion of inflammatory mediators and pro-inflammatory cytokines: (1) I kappa B kinase (IκB)/nuclear factor kappa B (NF-κB); (2) mitogen-activated protein kinase (MAPK); (3) phosphoinositide 3-kinase; and (4) Janus kinase (JAK) signal transducer and activator of transcription (STAT) signaling pathways [10,11,12].

Drug repurposing, or repositioning, is an attractive and practical approach to drug discovery that has demonstrated success in health care for years. The use of existing drugs in new indications is also a promising strategy for treating chronic inflammatory diseases as it can significantly reduce the development costs and time compared with de novo drug discovery [13]. Drug repurposing studies can be divided into two main categories, according to whether their approach is screening-based or mechanism-based. In the screening-based approach, the approved drugs are explored to discover new actions in the treatment or prevention of chronic inflammation whereas in the mechanism-based approach, drugs are screened for the confirmation of action against specific molecular targets. However, the search for novel therapies in the management of chronic inflammation continues, as the current treatments with these repositioned drugs are associated with one or more side effects [13,14].

**Figure 1:**
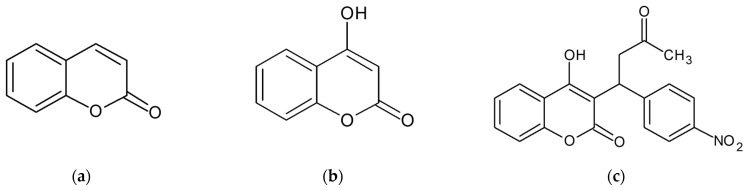
Structure of coumarin derivatives: (**a**) coumarin, (**b**) 4-hydroxycoumarin, and (**c**) acenocoumarol.

Herein, we examine the applicability of the anticoagulant acenocoumarol in drug repositioning as an anti-inflammatory agent. As shown in Figure 1, 4-hydroxycoumarin is a structural fragment of various natural and synthetic compounds, exhibiting various pharmacological activities. Its derivatives are attracting attention because of their properties as oral anticoagulants or rodenticides, photosensitizers, anti-HIV agents, and antibiotics. There has been a constant interest in the synthesis of these compounds [15,16,17]. In our efforts of drug repurposing with potent and safe skin health effects, we focused on the anticoagulants acenocoumarol to elucidate the anti-inflammatory properties of acenocoumarol using a RAW 264.7 macrophage model.

## 2. Results

### 2.1. Effects of Acenocoumarol on Viability, Pro-Inflammatory Mediators, and Cytokines of RAW 264.7 Cells

**Figure 2:**
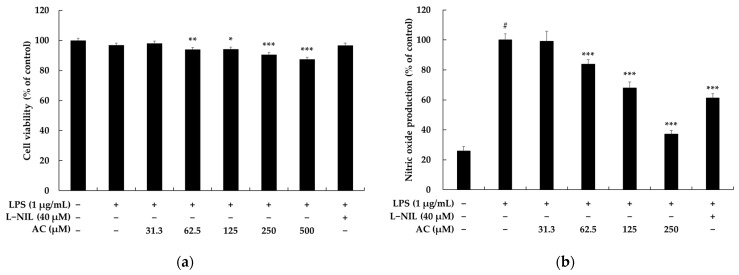
The effect of acenocoumarol on viability and nitric oxide (NO) production in RAW 264.7 cells. The cells were treated with acenocoumarol (AC, 31.3, 62.5, 125, 250, and 500 μM) and stimulated with LPS (1 μg/mL) for 24 h. L-NIL (40 μM) was used as the positive control. AC was evaluated using the MTT assay. Cell viability was expressed as a percentage relative to the untreated cells (**a**). The amount of NO in the medium was measured using the Griess reagent (**b**). The results are presented as the mean ± SD from three repeated experiments. # *p* < 0.001 vs. the unstimulated control group. * *p* < 0.05, ** *p* < 0.01, *** *p* < 0.001 vs. LPS alone.

**Figure 3:**
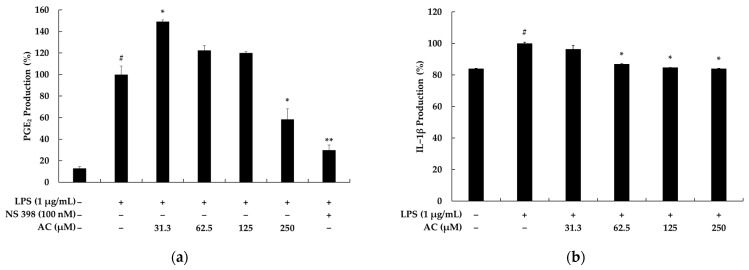

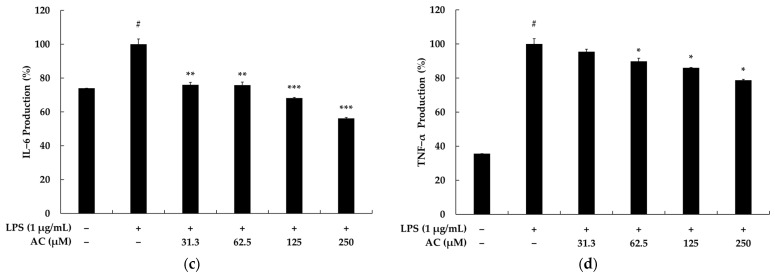
The effect of acenocoumarol on the production of PGE_2_ and pro-inflammatory cytokines in the LPS-induced RAW 264.7 cells. The cells were treated with acenocoumarol (31.3, 62.5, 125, and 250 μM) and LPS (1 μg/mL) stimulation for 24 h. NS 398 (100 nM) was used as the positive control. The production of (**a**) PGE_2_, (**b**) IL-1β, (**c**) IL-6, and (**d**) TNF-α were assessed using an ELISA kit. The results are presented as the mean ± SD from two repeated experiments. # *p* < 0.001 vs. the unstimulated control group. * *p* < 0.05, ** *p* < 0.01, *** *p* < 0.001 vs. LPS alone.

To investigate whether acenocoumarol exerts cytotoxicity on mouse macrophage RAW 264.7 cells, the cells were treated with various concentrations (31.3. 62.5, 125, 250, and 500 μM) of acenocoumarol and lipopolysaccharide (LPS) (1 μg/mL) for 24 h. The results show that there were no significant differences up to a 250 μM concentration of acenocoumarol in the RAW 264.7 cells (Figure 2a). Compared with those in the normal cells, the survival rates in the above samples were 98.06%, 93.92%, 94.14%, 90.58%, and 87.34%, respectively. Therefore, we used acenocoumarol concentrations of 31.3, 62.5, 125, and 250 μM in the subsequent experiments [18]. Nitric oxide (NO) is considered as an important mediator of increased production in cases of inflammation. Therefore, the suppression of excessive NO production is commonly used to evaluate the anti-inflammatory effects of compounds. NO production levels in the supernatants of RAW 264.7 macrophages activated by LPS are generally investigated using the Griess reagent. Figure 2b shows that in comparison with the control group, NO production was higher in the LPS-treated group. Acenocoumarol inhibits NO synthesis in LPS-activated RAW264.7 macrophages in a concentration-dependent manner, and the IC_50_ value was 191.62 ± 9.21 μM. L-N6-(1-iminoethyl)lysine (L-NIL), known to represent a potent inhibitor of inducible nitric oxide synthase, was used as a positive control. We next investigated whether acenocoumarol inhibits prostaglandin (PG)E_2_ and inflammatory cytokines in the LPS-stimulated RAW 264.7 cells. Our results show that acenocoumarol inhibits PGE_2_, interleukin (IL)-6, IL-1β, and tumor necrosis factor (TNF)-α production in a concentration-dependent manner (Figure 3).

### 2.2. Effect of Acenocoumarol on NO Synthase (iNOS) and Cyclooxygenase (COX)-2 Production

**Figure 4:**
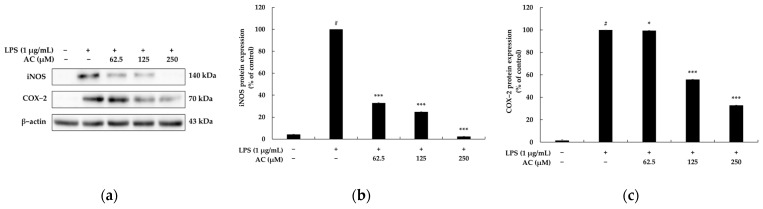
The effect of acenocoumarol on iNOS and COX-2 protein expression in the LPS-induced RAW 264.7 cells. The cells were treated with acenocoumarol (62.5, 125, and 250 μM) and LPS (1 μg/mL) stimulation for 22 h. (**a**) Western blotting results, (**b**) iNOS protein expression, (**c**) COX-2 protein expression. The results are presented as the mean ± SD from three repeated measurements using ImageJ. # *p* < 0.001 vs. the unstimulated control group. * *p* < 0.05, *** *p* < 0.001 vs. LPS alone.

Inducible NO synthase and COX-2 are strongly linked to the regulation of NO and PGE_2_ production during inflammatory responses. Hence, many researchers have tried to find new compounds that can inhibit the production of these enzymes [19]. To understand the mechanism by which acenocoumarol inhibits NO and PGE_2_ production, the abundance of iNOS and COX-2 proteins was evaluated using Western blotting. As shown in Figure 4, iNOS production was significantly decreased in a concentration-dependent manner by acenocoumarol (*p* < 0.05), decreasing by 67.00, 75.13, and 97.60% at 62.5, 125, and 250 μM of acenocoumarol, respectively. Furthermore, the COX-2 levels were significantly reduced after the exposure of LPS-induced RAW264.7 to acenocoumarol, also in a concentration-dependent manner (62.5, 125, and 250 μM), resulting in decreases of 0.54, 44.16, and 67.09%, respectively, compared with the activated macrophages. The expression of iNOS and COX-2 was not affected when unstimulated RAW 264.7 cells were treated only with acenocoumarol. After stimulation, the macrophages produced increased amounts of iNOS and COX-2, indicating that the pro-inflammatory response is directly related to the production of NO and PGE_2_.

### 2.3. Effect of Acenocoumarol on the Mitogen-Activated Protein Kinase (MAPK) Signaling Pathway

**Figure 5:**
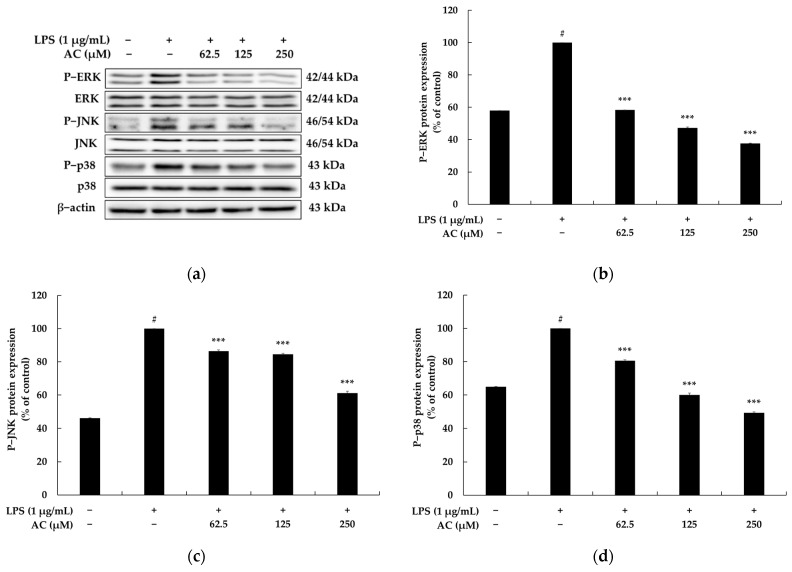
The effect of acenocoumarol on MAPK protein expression in the LPS-induced RAW 264.7 cells. The cells were treated with acenocoumarol (62.5, 125, and 250 μM) and LPS (1 μg/mL) stimulation for 20 min. (**a**) Western blotting results, (**b**) P-ERK protein expression, (**c**) P-JNK protein expression, (**d**) P-p38 protein expression. The results are presented as the mean ± SD from three repeated measurements using Image J. # *p* < 0.001 vs. the unstimulated control group. *** *p* < 0.001 vs. LPS alone.

It has been reported that the phosphorylation of MAPK, consisting of three pathways involving extracellular-signal-regulated kinase 1 and 2 (ERK1/2), c-Jun N-terminal kinase 1, 2, and 3 (JNK1/2/3), and p38 MAPK activates signaling pathways and increases the production of various inflammatory cytokines. This pathway is reportedly involved in inflammatory cytokine expression, cell growth, cell proliferation, cell differentiation, stress response, migration, and apoptosis and is activated in response to various extracellular stimuli (KK). Overall, the MAPK signaling pathway plays a crucial role in regulating inflammatory cytokines in an inflammatory response. To investigate the relationship between acenocoumarol function and MAPK pathways, the phosphorylation levels of ERK, JNK, and p38 were evaluated in the LPS-stimulated macrophages exposed to acenocoumarol. As presented in Figure 5, the phosphorylation levels of ERK, JNK, and p38 increased significantly after LPS treatment in the vehicle + LPS group compared with the untreated group. However, the administration of acenocoumarol to the LPS-stimulated RAW264.7 cells significantly inhibited the phosphorylation of ERK, JNK, and p38 compared with the vehicle + LPS group. Even acenocoumarol treatment remarkably decreased the level of phosphorylated ERK and p38 compared with the LPS treatment at 125 μM and 250 μM concentrations. Taken together, our results suggest that acenocoumarol suppresses the LPS-induced inflammatory response through the regulation of the iNOS-mediated COX-2-induced pathway, inflammatory cytokine transcription, and the MAPK signaling pathway.

### 2.4. Effect of Acenocoumarol on the Nuclear Factor κB (NF-κB) Signaling Pathway

**Figure 6:**
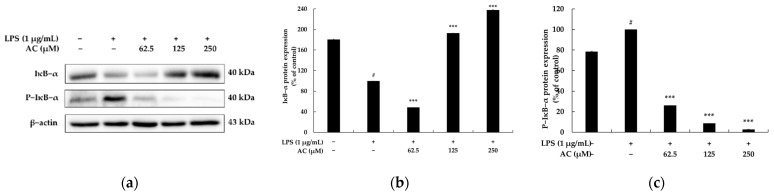
The effect of acenocoumarol on IκBα and P-IκBα protein expression in the LPS-induced RAW 264.7 cells. The cells were treated with acenocoumarol (62.5, 125, and 250 μM) and LPS (1 μg/mL) stimulation for 20 min. (**a**) Western blotting results, (**b**) IκBα protein expression, (**c**) P-IκBα protein expression. The results are presented as the mean ± SD from three repeated measurements using ImageJ. # *p* < 0.001 vs. the unstimulated control group. *** *p* < 0.001 vs. LPS alone.

**Figure 7:**
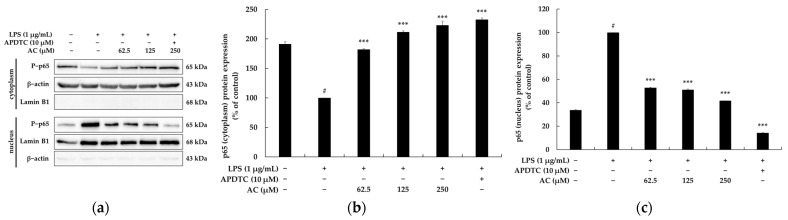
The effect of acenocoumarol on the NF-κB (p65) protein expression in the LPS-induced RAW 264.7 cells. The cells were treated with acenocoumarol (62.5, 125, and 250 μM) and LPS (1 μg/mL) stimulation for 15 min. (**a**) Western blotting results, (**b**) p65 (cytoplasm) protein expression, (**c**) p65 (nucleus) protein expression. Extracts were assessed for nuclear purity by the expression of lamin B1. The purity of the cytoplasmic extracts was verified by the β-actin levels. Results indicate minimal cross-contamination of the nuclear and cytoplasmic extracts. The results are presented as the mean ± SD from three repeated measurements using ImageJ. # *p* < 0.001 vs. the unstimulated control group. *** *p* < 0.001 vs. LPS alone.

It has been reported that when macrophages are stimulated with LPS, IκBα is phosphorylated and ubiquitinated. Accordingly, phosphorylated NF-κB is translocated from the cytoplasm to the nucleus to increase the levels of inflammatory cytokines. As above-mentioned, aspirin and sodium salicylate block the degradation of IκBα, thus inhibiting the migration of NF-κB into the nucleus, thereby showing anti-inflammatory effects [20]. Therefore, Western blotting experiments were performed to investigate whether acenocoumarol inhibits the production of inflammatory cytokines through the NF-κB signaling pathway in LPS-stimulated RAW 264.7 cells. As shown in Figure 6, the levels of p-p65/β-actin and p-IκBα/β-actin phosphorylated proteins were markedly upregulated by LPS stimulation. Conversely, the IκBα/β-actin protein levels were downregulated in acenocoumarol-treated RAW 264.7 cells. We further investigated whether acenocoumarol could dampen the NF-κB p65 nuclear translocation in the LPS-stimulated RAW264.7 cells. As shown in Figure 7, the nuclear translocation of p65 was increased by LPS stimulation. However, following pre-treatment with acenocoumarol, p65 nuclear translocation was profoundly decreased compared with the model group. The purity of the nuclear and cytoplasmic extracts was verified by probing the blots with cytoplasmic protein β-actin and nuclear protein lamin B1. Minimal cross-contamination of the fractions was seen. APDTC also blocked NF-κB translocation as a positive control. In the presence of APDTC (10 μM), the levels of the nuclear NF-KB induced by LPS were decreased in the RAW 264.7 cells, confirming the involvement of NF-KB in the activation of RAW264.7 cells by acenocoumarol. Overall, this experimental work documents that acenocoumarol inhibits the LPS-stimulated p65 nuclear translocation and the NF-κB signaling pathway.

## 3. Discussion

Developing a new drug takes more than 10 years because it goes through various processes such as target discovery, the screening and optimization of candidates, evaluation of pharmacological and pharmacokinetics, and formulation. However, drug repurposing is the process of registering a new use for an approved drug that can be achieved within three years, which is much faster than the drug development period. In the course of the drug-repurposing strategy for the development of functional components, we have reported new applications of many FDA-approved existing drugs such as tobramycin, lincomycin, and fosfomycin to enhance the melanogenic effects [21,22,23], and spiramycin and D-cycloserine for exerting anti-inflammatory effects [24,25].

Acenocoumarol is not approved for marketing in the United States by the U.S. Food and Drug Administration, but is available in Canada and other countries. Acenocoumarol is a 4-hydroxycoumarin derivative used as an anticoagulant in the prevention of thromboembolic diseases in infarction and transient ischemic attacks as well as in the management of deep vein thrombosis and myocardial infarction. In addition, acenocoumarol also dose-dependently inhibits tryptophan breakdown in IFN-γ-stimulated Caco-2 cells [26,27]. Warzecha et al. found that low doses of acenocoumarol, given before the induction of acute pancreatitis by cerulein, inhibited the development of that inflammation [28]. Furthermore, treatment with acenocoumarol accelerates the recovery of ischemia/reperfusion-induced acute pancreatitis in rats [29]. On the other hand, 4-hydroxycoumarin itself is not an anticoagulant, but it has anticoagulant activity when a large aromatic substituent is added to the 3-position (the ring-carbon between the hydroxyl and the carbonyl). Large 3-position substituents are required for anticoagulant activity [30]. As is well-known, aenocoumarol has anticoagulant activity in the form of substituted α-acetonyl-ρ-nitrobenzyl structure at position 3 of 4-hydroxy-coumarin. Other than position 3, simple 4-hydroxycoumarin derivatives have been reported to have some anti-inflammatory effects, but they are difficult to use for drugs or skin damage due to the side effects. Previous studies have shown that among the natural coumarins, several compounds such as psoralen, bergapten, and xanthotoxin evoked a limited number of dermal phototoxic reactions in humans. It also showed that coumarins, especially their 3,4-epoxide intermediates, could induce vacuolar degeneration, necrosis, and hepatocyte apoptosis in rat liver [31]. As an extension of these studies, we therefore investigated the possibility of repurposing acenocoumarol as an anti-inflammatory agent.

RAW 264.7, a murine macrophage cell line, is an excellent model for anti-inflammatory drug screening and for subsequently evaluating the inhibitors of pathways leading to the induction of pro-inflammatory cytokines. The present study was undertaken to elucidate the pharmacological effects and mechanisms of acenocoumarol on the production of inflammatory mediators and pro-inflammatory cytokines in the RAW 264.7 cells stimulated with LPS in vitro.

First, the anti-inflammatory effects of acenocoumarol were investigated on the RAW264.7 cells stimulated with LPS (1 μg/mL), and we determined the nontoxic concentrations of acenocoumarol with or without LPS in the RAW264.7 macrophages using a MTT viability assay. The decrease in RAW264.7 cell viability following LPS treatment due to the release of inflammatory substances, which may act as cytotoxic agents, has been previously demonstrated, but an increase in the cell viability or little change in cell viability has also been reported [32,33,34,35]. However, in our MTT assay, LPS (1 μg/mL) treatment of the RAW264.7 cells was associated with a slight decrease in their viability compared with the nontreated cells or cells treated with acenocoumarol alone. Acenocoumarol did not decrease the cell viability at 500 μM; therefore, acenocoumarol was used at 62.5, 125, and 250 μM in the efficacy study.

Furthermore, we evaluated the production of NO and PGE2 as important inflammatory biomarkers responsible for rubefaction, pain, fever, swelling, and malfunction. Our data indicate that the upregulated secretion of NO and PGE2 by LPS in the RAW 264.7 macrophages was progressively inhibited by acenocoumarol in a concentration-dependent manner, and this is related to the inhibition of iNOS and COX-2 protein expression. These results indicate that the anti-inflammatory effect of acenocoumarol is at least due to the reduced expression of iNOS and COX-2, which are required for NO and PGE2 production.

Pro-inflammatory cytokines are mutually inducible and have a dual role of inhibiting or promoting the progression of inflammation [36]. TNF-α can be upregulated to an appropriate amount and restored to enhance immune effects, whereas excessive amounts of TNF-α can easily induce the release of IL-6 and other related inflammatory cytokines. IL-1β is secreted by monocytes and macrophages, and it should be noted that IL-1β acts cooperatively with IL-6 to generate a cascade effect that exacerbates the inflammatory process. It has been reported that the LPS treatment of RAW264.7 macrophages significantly increases the release of pro-inflammatory cytokines such as TNF-α, IL-6, and IL-1β. Therefore, the levels of pro-inflammatory cytokines are applied as an indicator to evaluate the anti-inflammatory efficacy of macrophages [37,38]. In this study, we found that acenocoumarol inhibits the production of TNF-α, IL-1β, and IL-6 in the LPS-induced RAW 264.7 macrophages by suppressing their expression.

To elucidate the underlying mechanism responsible for the anti-inflammatory effects of acenocoumarol, its regulatory effects on the NF-κB inflammatory signaling pathway were investigated in RAW 264.7 cells stimulated by LPS. Numerous studies have reported that NF-κB is a key transcription factor in the pathogenesis of inflammatory diseases, and its activation positively regulates the expression of inflammatory mediators (e.g., iNOS and COX-2) and pro-inflammatory cytokines (e.g., TNF-α, IL-1β, and IL-6). In addition, NF-κB mainly consists of p50 and p65 subunits, the latter responding to pre-inflammatory cytokine stimulation [6,19,20]. NF-κB is a cytosolic inactive form that binds to unphosphorylated IκB in a steady state. Under inflammatory stimuli such as LPS, active IKK phosphorylates IκBα, followed by the phosphorylation and then ubiquitination of IκBα, which is subsequently degraded by the 26S proteasome. After IκBα degradation, cytoplasmic NF-κB migrates to the nucleus and binds to the target gene, resulting in the transcription of inflammatory genes [39]. To establish whether NF-κB mediates anti-inflammatory processes in response to acenocoumarol, IκBα phosphorylation and NF-κB p65 translocation were evaluated by Western blotting. Acenocoumarol was found to inhibit the phosphorylation of the IκBα protein and suppress LPS-induced NF-κB (p65) translocation. Thus, acenocoumarol inhibits the NF-κB signaling pathway to alleviate LPS-induced inflammation.

Mitogen-activated protein kinase (MAPK) is another important signaling pathway activated in LPS-induced macrophages through the TLR4. During the inflammation process, members of the MAPK family, consisting of three main subfamily members (i.e., ERK, p38, and JNK), are phosphorylated to adjust the expression of iNOS, COX-2, and other pro-inflammatory cytokines. Thus, it has been suggested that inhibitors targeting the p38 and JNK MAPK pathways have anti-inflammatory activity [40,41]. To investigate whether the MAPK signaling pathway is also affected by acenocoumarol, the phosphorylation of ERK, p38, and JNK was analyzed. The results indicate that the levels of phosphorylated ERK, p38, and JNK in the RAW 264.7 macrophages are significantly increased in the presence of LPS, while treatment with acenocoumarol results in a concentration-dependent decrease in the phosphorylation of the MAPK family proteins. These results suggest that the effects of acenocoumarol on the production of inflammatory mediators and pro-inflammatory cytokines are likely mediated through the blocking of the ERK, p38, and JNK signaling pathways in the RAW 264.7 cells.

## 4. Materials and Methods

### 4.1. Materials

The acenocoumarol used in this study was purchased from TCI (Tokyo, Japan), and warfarin was purchased from Sigma-Aldrich (St. Louis, MO, USA). For cell culture, Dulbecco’s modified Eagle’s medium (DMEM) and penicillin–streptomycin (P/S) were purchased from Thermo Fisher Scientific (Waltham, MA, USA) and fetal bovine serum (FBS) from Merck Millipore (Burling, MA, USA). Protease/phosphatase inhibitor cocktail, sodium hydroxide (NaOH), lipopolysaccharide (LPS), and Griess reagent used for the cell experiments were purchased from Sigma-Aldrich (St. Louis, MO, USA), 3-(4,5-dimethylthiazol-2-yl)-2,5-diphenyltetrazolium bromide (MTT), dimethyl sulfoxide (DMSO), phosphate-buffered saline (PBS), Tris-buffered saline (TBS), sodium dodecyl sulfate (SDS), radioimmunoprecipitation assay (RIPA) buffer, and an enhanced chemiluminescence (ECL) kit were purchased from Biosesang (Seongnam, Gyeonggi-do, Republic of Korea). A BCA protein assay kit, NE-PER nuclear and cytoplasmic extraction reagents, and 0.5% trypsin–ethylenediaminetetraacetic acid (10×) were purchased from Thermo Fisher Scientific (Waltham, MA, USA), and Tween 20 and 2× Laemmli sample buffer were obtained from Bio-Rad (Hercules, CA, USA). Skim milk was purchased from BD Difco (Sparks, MD, USA). Among the enzyme-linked immunosorbent assessment (ELISA) kits, PGE_2_ was purchased from abcam (Cambridge, EN, UK), and IL-1β, IL-6, and TNF-α from BD Biosciences (Franklin Lakes, NJ, USA). COX-2 was purchased from BD Biosciences (Franklin Lakes, NJ, USA) and p-ERK (9101S), ERK (9102S), p-p38 (9211S), p38 (9212S), p-JNK (9251S), JNK (9252S), iNOS (2982S), p-IκBα (9246S), IκBα (4812S), p65 (4764S), lamin B (12586), β-actin (4967S), and secondary anti-mouse and anti-rabbit antibodies were purchased from Cell Signaling Technology (Danvers, MA, USA).

### 4.2. Cell Culture

The RAW 264.7 murine macrophage cells were purchased from the Korea Cell Line Bank (Seoul, Republic of Korea). Cells were cultured in Dulbecco’s modified Eagle’s medium (DMEM) with 10% fetal bovine serum (FBS) and 1% penicillin–streptomycin at 37 °C in a humidified 5% CO_2_ atmosphere.

### 4.3. MTT Assay

Cytotoxicity was assessed using the MTT assay. Cultured RAW 264.7 cells (1.5 × 10^5^ cells/well) were treated with coumarin derivatives and LPS (1 μg/mL) in 24-well plates and incubated for 24 h. For the MTT assay, the culture medium was replaced with 0.2 mg/mL MTT (500 μL). The cells were incubated at 37 °C for 3 h, the medium was removed, and the formazan product was dissolved in dimethyl sulfoxide. Absorbance was measured at 570 nm using a microplate reader (Biotek; Winooski, VT, USA).

### 4.4. Measurement of NO Production

The NO concentration in the culture supernatants was measured in the form of nitrite in the cell culture medium using the Griess reagent. Cultured RAW 264.7 cells (1.5 × 10^5^ cells/well) were treated with acenocoumarol and LPS (1 μg/mL) in 24-well plates and incubated for 24 h. As a positive control group, iNOS-specific inhibitor L-NIL (40 μM) was used. Cell culture supernatants were mixed with an equal volume (100 μL) of the Griess reagent and incubated in 96-well plates for 10 min at room temperature. Absorbance was measured at 540 nm using a microplate reader (Biotek; Winooski, VT, USA).

### 4.5. Measurement of PGE_2_ and Cytokines

The levels of PGE_2_ and cytokines (IL-1β, IL-6, and TNF-α) in the culture supernatants were determined using cytokine detection ELISA kits, as per the manufacturer’s instructions. Cultured RAW 264.7 cells (1.5 × 10^5^ cells/well) were treated with acenocoumarol and LPS (1 μg/mL) in 24-well plates and incubated for 24 h. As a positive control when measuring PGE_2_ production, the COX-2 specific inhibitor NS 398 (100 nM) was used. Protein levels were determined by measuring absorbance at 405 or 450 nm using a microplate reader (Biotek; Winooski, VT, USA).

### 4.6. Preparation of Nuclear and Cytoplasmic Extraction

Nuclear and cytoplasmic extracts were isolated using an extraction kit. The RAW 264.7 cells (6.0 × 10^5^ cells/dish) were incubated in 60 mm cell culture dishes for 24 h. Acenocoumarol and LPS (1 μg/mL) were treated and cultured to determine the expression of each protein. After incubation, a nuclear extract was obtained according to the protocols provided by the manufacturer of the extraction kit.

### 4.7. Western Blotting

The RAW 264.7 cells (6.0 × 10^5^ cells/dish) were incubated in 60 mm cell culture dishes for 24 h. Acenocoumarol and LPS (1 μg/mL) were treated and cultured for each protein expression time. The cells were washed with 1× PBS, and lysis buffer (RIPA buffer, 1% protease inhibitor cocktail) was added for lysis at 4 °C for 20 min. Supernatants were obtained after centrifugation at 15,000 rpm for 20 min at −8 °C. The protein concentration was quantified using a BCA protein assay kit and adjusted to 30 μg/mL. For loading onto gels, samples of the protein and 2× Laemmli sample buffer were mixed in a 1:1 ratio and heated at 100 °C for 5 m. Samples were electrophoresed on an SDS–polyacrylamide gel for size separation of the proteins. After transferring the proteins to a PVDF membrane, the membrane was blocked in 5% skim milk dissolved in TBS-T (Tris-buffered saline with 1% Tween 20) for 2 h. The membrane was washed with 1× TBS-T and incubated with primary antibody diluted at a ratio of 1:2000 overnight at 4 °C. After washing the antibody, the membrane was incubated with secondary antibody diluted at a ratio of 1:1000 at room temperature for 2 h. After washing the antibody, the signal was developed using an ECL kit and measured using Chemidoc (VILBER LOURMAT, Marne La Vallée, France).

### 4.8. Statistical Analyses

The results of the experiments were expressed as the mean and standard deviation (mean ± SD) through three repeated experiments. Statistical significance was determined based on *p*-values using the Student’s *t*-test, # *p* < 0.001 vs. the unstimulated control group. * *p* < 0.05, ** *p* < 0.01, *** *p* < 0.001 vs. LPS alone.

## 5. Conclusions

**Figure 8:**
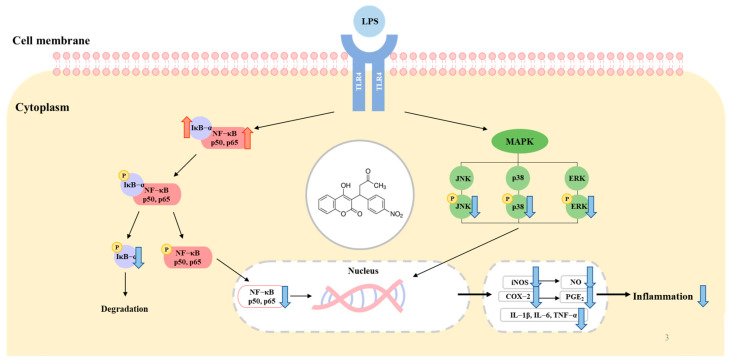
A schematic of the proposed mechanism of acenocoumarol in inhibiting LPS-stimulated inflammation in the RAW264.7 cells. Acenocoumarol inhibits the MAPK and NF-κB pathway.

After collecting all of the information regarding the anti-inflammatory effects of acenocoumarol identified in this study, a map of the relevant molecular pathways was constructed (Figure 8). First, acenocoumarol inhibited iNOS and COX-2 expression, indicating that the pro-inflammatory response is directly related to the production of NO and PGE_2_ in the LPS-induced RAW 264.7 cells. Second, acenocoumarol inhibited interleukin IL-6, IL-1β, and TNF-α production in a concentration-dependent manner. Finally, acenocoumarol exhibited anti-inflammatory activity that depends on its ability to regulate the production of NO, PGE_2_, and other pro-inflammatory cytokines in the LPS-induced RAW 264.7 cells through the suppression of NF-κB activation and MAPK phosphorylation. Considering these results, acenocoumarol can be considered as a possible candidate for repurposing as an anti-inflammatory agent. However, further studies are needed to fully understand the role of cellular signaling pathways other than the MAPK and NF-κB pathways, which may be involved in the anti-inflammatory activity of acenocoumarol. Furthermore, the mechanisms involved in the anti-inflammatory efficacy of acenocoumarol should be assessed with another cell line and in vivo.

## Data Availability

Not applicable.
